# How fear and collectivism influence public’s preventive intention towards COVID-19 infection: a study based on big data from the social media

**DOI:** 10.1186/s12889-020-09674-6

**Published:** 2020-11-16

**Authors:** Feng Huang, Huimin Ding, Zeyu Liu, Peijing Wu, Meng Zhu, Ang Li, Tingshao Zhu

**Affiliations:** 1 Institute of Psychology, Chinese Academy of Sciences, Beijing, 100101 China; 2 Department of Psychology, University of Chinese Academy of Sciences, Beijing, 100049 China; 3 Graduate School, Xinyang Normal University, Xinyang, 464000 China; 4 Institute for Advanced Studies in Finance and Economics, Hubei University of Economics, Wuhan, 430205 China; 5 Department of Psychology, Beijing Forestry University, Beijing, 100101 China

**Keywords:** COVID-19, Prevention and control, Fear, Cultural characteristics, Social media, Big data analysis

## Abstract

**Background:**

Despite worldwide calls for precautionary measures to combat COVID-19, the public’s preventive intention still varies significantly among different regions. Exploring the influencing factors of the public’s preventive intention is very important to curtail the spread of COVID-19. Previous studies have found that fear can effectively improve the public’s preventive intention, but they ignore the impact of differences in cultural values. The present study examines the combined effect of fear and collectivism on the public’s preventive intention towards COVID-19 through the analysis of social media big data.

**Methods:**

The Sina microblog posts of 108,914 active users from Chinese mainland 31 provinces were downloaded. The data was retrieved from January 11 to February 21, 2020. Afterwards, we conducted a province-level analysis of the contents of downloaded posts. Three lexicons were applied to automatically recognise the scores of fear, collectivism, and preventive intention of 31 provinces. After that, a multiple regression model was established to examine the combined effect of fear and collectivism on the public’s preventive intention towards COVID-19. The simple slope test and the Johnson-Neyman technique were used to test the interaction of fear and collectivism on preventive intention.

**Results:**

The study reveals that: (a) both fear and collectivism can positively predict people’s preventive intention and (b) there is an interaction of fear and collectivism on people’s preventive intention, where fear and collectivism reduce each other’s positive influence on people’s preventive intention.

**Conclusion:**

The promotion of fear on people’s preventive intention may be limited and conditional, and values of collectivism can well compensate for the promotion of fear on preventive intention. These results provide scientific inspiration on how to enhance the public’s preventive intention towards COVID-19 effectively.

## Background

As a global public health crisis, COVID-19 has become an unprecedented situation with World War characteristics [1]. Since a specific vaccine has not yet been approved for use, non-drug prophylaxis has been the leading alternative used in blocking the spread of COVID-19. The preventive behaviours (e.g. wearing masks and reducing aggregation) are effective in reducing the spread of COVID-19 [2, 3]. However, despite calls from the government and the media for people to take steps in the prevention of COVID-19, there are still significant differences in the preventive behaviours of people in different regions [4, 5]. Identifying the determinants of the public’s intention in the prevention of COVID-19 is essential. Such would be the determinants of reducing the spread of COVID-19.

Existing studies of COVID-19 have focused on epidemiology and mental health, with the former being devoted to analysing the epidemiological characteristics of the virus [6, 7], Conversely, the latter focused on the psychological consequences of the pandemic [8, 9]. Few researchers have focused on the public’s intention to prevent the COVID-19 pandemic and its psychological mechanisms. Previous studies have found that it was functional fear, rather than risk perception [10], moral foundations [11] and political orientation [12] that positively predicted people’s preventive intention towards COVID-19 [13]. In contrast, another study has shown that the high level of personal prevention measures (e.g., wash hands after cough) were associated with the low level of psychiatric symptoms such as less depression, anxiety and stress [14]. The difference with other negative psychology is that fear could encourage public’s preventive behaviours towards COVID-19 infection. However, we have noticed that despite the widespread fear in the face of COVID-19 [15], the regional differences in public’s preventive intention persist [5]. As a common human emotion that arises spontaneously in the face of danger [16], fear does not fully explain the significant differences in the behaviour of people during COVID-19. Predicting people’s preventive intention through fear alone is not enough.

The inclusion of cultural values variables shall aid in elucidating the issues mentioned above. Individualism and collectivism are two distinct cultural values [17], individualism value personal autonomy, uniqueness and independence while collectivism value person-other relatedness or interdependence and person as being part of a collective [18]. Although individualism-collectivism is often used to compare cultures, they are also used as regions and individuals differences variable within a culture [19]. As far as China is concerned, previous studies have found that collectivism in the south is generally higher than that in the north [20]. More remarkable, In a very collectivistic society such as China, some members can be as individualistic as those in the United States, and vice versa [21]. The pathogen prevalence hypothesis holds that collectivism is more likely to promote protection against epidemics than does individualism [22, 23]. As collectivism places more emphasis on in-group vigilance [24–26], such may contribute to people’s intention to prevent COVID-19.

Furthermore, different cultural values may lead to different emotional responses [27], previous research has found that the collectivism enhances the effectiveness of people’s psychological protection and thus buffers the impact of negative emotions on people [28]. Based on the insights from the pathogen epidemic hypothesis, we predict that the collectivism degree will raise people’s preventive intention. It shall likewise diminish the impact of fear on their preventive intention during the COVID-19 pandemic.

The emotions, cultural values and behavioural intentions are usually measured by retrospective questionnaires, such as the Positive and Negative Affect Schedule (PANAS) [29], the Individualism–Collectivism Scale (ICS) [30] and the Users’ Information Security Awareness Questionnaire (UISAQ) [31]. However, social isolation makes the use of paper questionnaires complicated during the COVID-19 pandemic. Online surveys rely on the cooperation of participants. Furthermore, it may lead to difficulty in meeting timely requirements and even bring extra burden for the participants [32]. During the COVID-19 pandemic, social isolation led to a significant increase in people’s social media exposure [33]. Such provided an excellent opportunity for people to use social media to study the psychological characteristics of other people. As a non-invasive analysis, the validity and superiority of using social media behaviour data (e.g. posts, comments and replies) to measure user’s emotions, cultural values and behavioural intentions have been proven [34–36].

In summary, the present study explores the joint impact of fear and collectivism on people’s preventive intention towards COVID-19, based on Sina Microblog big data from 31 regions in mainland China during the pandemic. The present study aims to provide useful recommendations for the prevention and control of COVID-19 from a psychological perspective.

## Methods

### Participants and data collection

Since the period from January 11 to February 21, 2020, was the worst period of the outbreak in mainland China [37, 38], we collected data from Sina Microblog in mainland China during this period. The samples in this study were from the original Microblog data pool containing more than 1.16 million active Microblog users [39]. Microblog users are firstly screened by the following requirements: (a) the registration period is more than one year; (b) non-public, commercial or robot accounts; (c) they had published at least ten original Microblog posts during January 11 to February 21, 2020. We acquired 108,914 active Microblog users across 31 provinces finally, then downloaded all their original posts published from January 11 to February 21, 2020.

### Calculation of psychological indicators

This study employed TextMind system developed by the Computational CyberPsychology Laboratory at the Institute of Psychology, Chinese Academy of Sciences to extract the 31 provinces’ Microblog content features [40]. The TextMind system provides an all-in-one solution from automatic Chinese words segmentation to calculating the frequency of specific keywords [39, 41]. The keywords lexicon of fear, collectivism, and preventive intention are as follows:

#### Fear

The *Weibo Five Basic Mood Lexicon (Weibo-5BML)* has been developed to measure the raw emotions of Sina Microblog users [42]. *Weibo-5BML* contains 72 fear-keywords, such as ‘Haipa’ (afraid), ‘Jinghuang’ (panic-stricken) and ‘Konghuang’ (scare), and its validity in measuring emotions has been repeatedly validated [34, 43].

#### Collectivism

*Individualism-Collectivism Lexicon* has been developed to measure the cultural values of social media users [35]. There are 53 individualism-keywords (e.g., ‘wo’, means I/me; ‘Jingzheng’, means competition) and 112 collectivism-keywords (e.g., ‘women’, means we/us; ‘Hezuo’, means cooperation).

#### Preventive intention of COVID-19

Following the previous studies [36, 44], this study selected the keyword related to COVID-19 prevention from the Sina Microblog during the outbreak. After discussion by the panel of experts, we get eight keywords in follow: ‘Daikouzhao’ (wear a mask), ‘Xiaodu’ (frequent disinfection), ‘Fangyi’ (epidemic prevention), ‘Bujuji’ (social distancing), ‘Bujuhui’ (refuse to the party), ‘Bujucan’ (refuse to dine together), ‘Buchumen’ (insist on staying home) and ‘Xishou’ (frequent hand-washing).

**Fig. 1:**
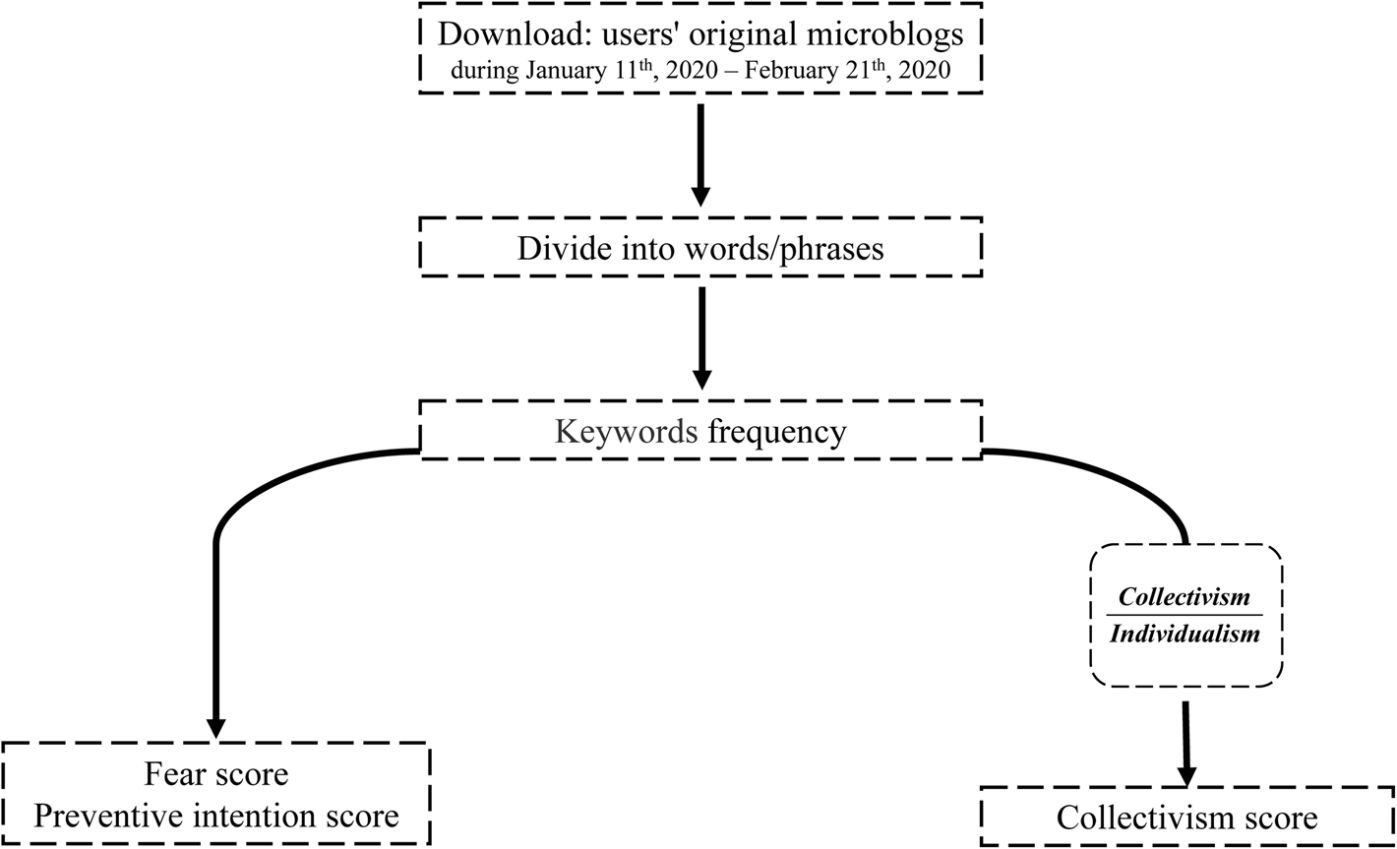
The procedure from data collection to calculation of psychological indicators

This study used specific keywords frequencies as the scores of fear and preventive intention on every province, used the ratio of collectivism word frequency to individualism word frequency as every provinces’ collectivism score. Figure 1 portrays the procedure from data collection to calculation of psychological indicators.

### Data analysis

SPSS 23.0 was used to establish the database and conduct preliminary statistical analysis. The interaction effect was tested by the *PROCESS for SPSS 3.3* [45].

Firstly, the *Pearson correlation* was calculated to test the relationship between variables. We then conducted a multiple regression analysis. Bootstrap method and Johnson-Neyman method [45, 46] were used to examine the interaction of fear and collectivism on preventive intention. Besides, to exclude the multicollinearity of independent variables, we took the variance inflation factor (VIF) to diagnose the regression model [47]. Results show that the VIF of all independent variables is not greater than 1.2.

### Ethical issues

These freely available posts were downloaded from Sina microblog. The personal privacy of users was strictly protected during the procedure. The names, IDs and original posts of all users do not appear in the present study as it only involves the analysis of provincial data. This research project was approved by the Ethics Committee, Institute of Psychology, Chinese Academy of Sciences (project number: H15009).

## Results

### Descriptive statistics and correlation coefficient

**Fig. 2:**
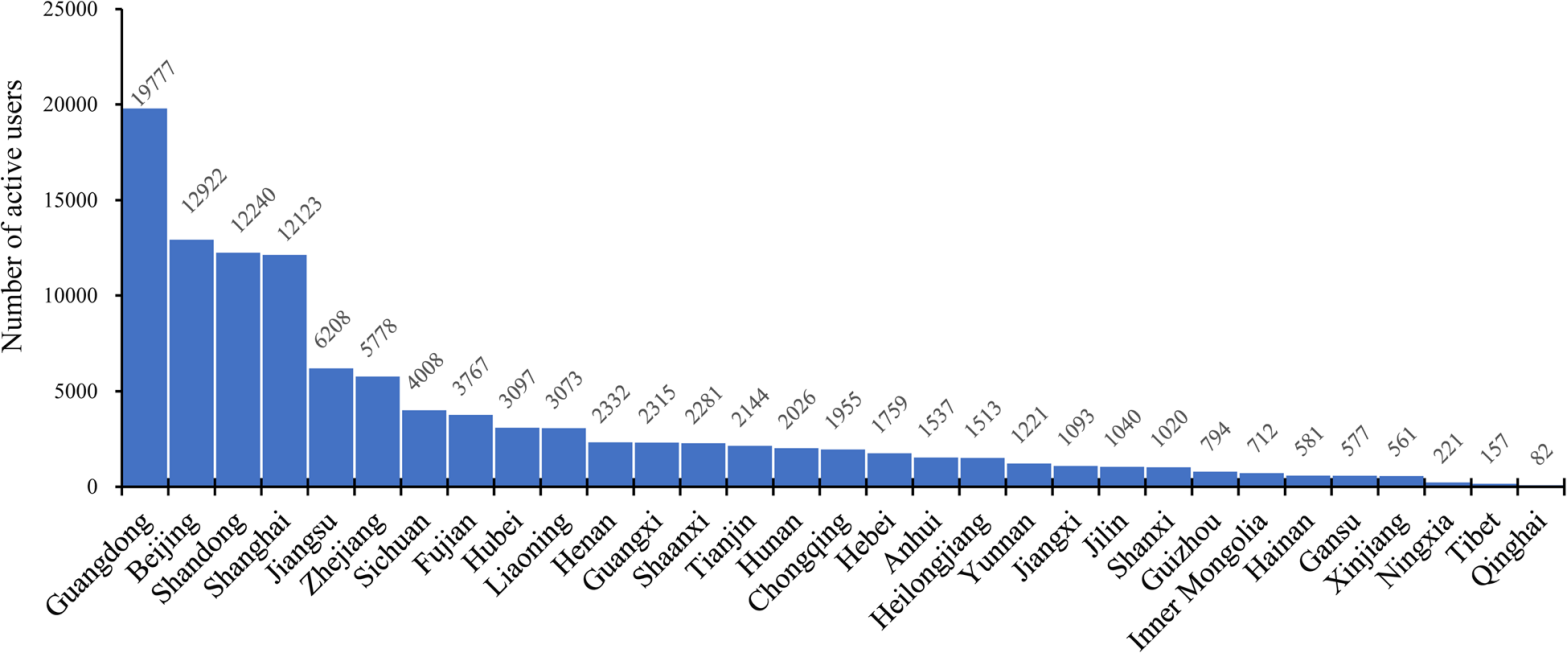
The regional distribution of active Microblog users during the COVID-19 period (number of people)

In this study, all provinces except Qinghai have more than 100 active users. The regional distribution of 108,914 active Microblog users is shown in Fig. 2.

**Fig. 3:**
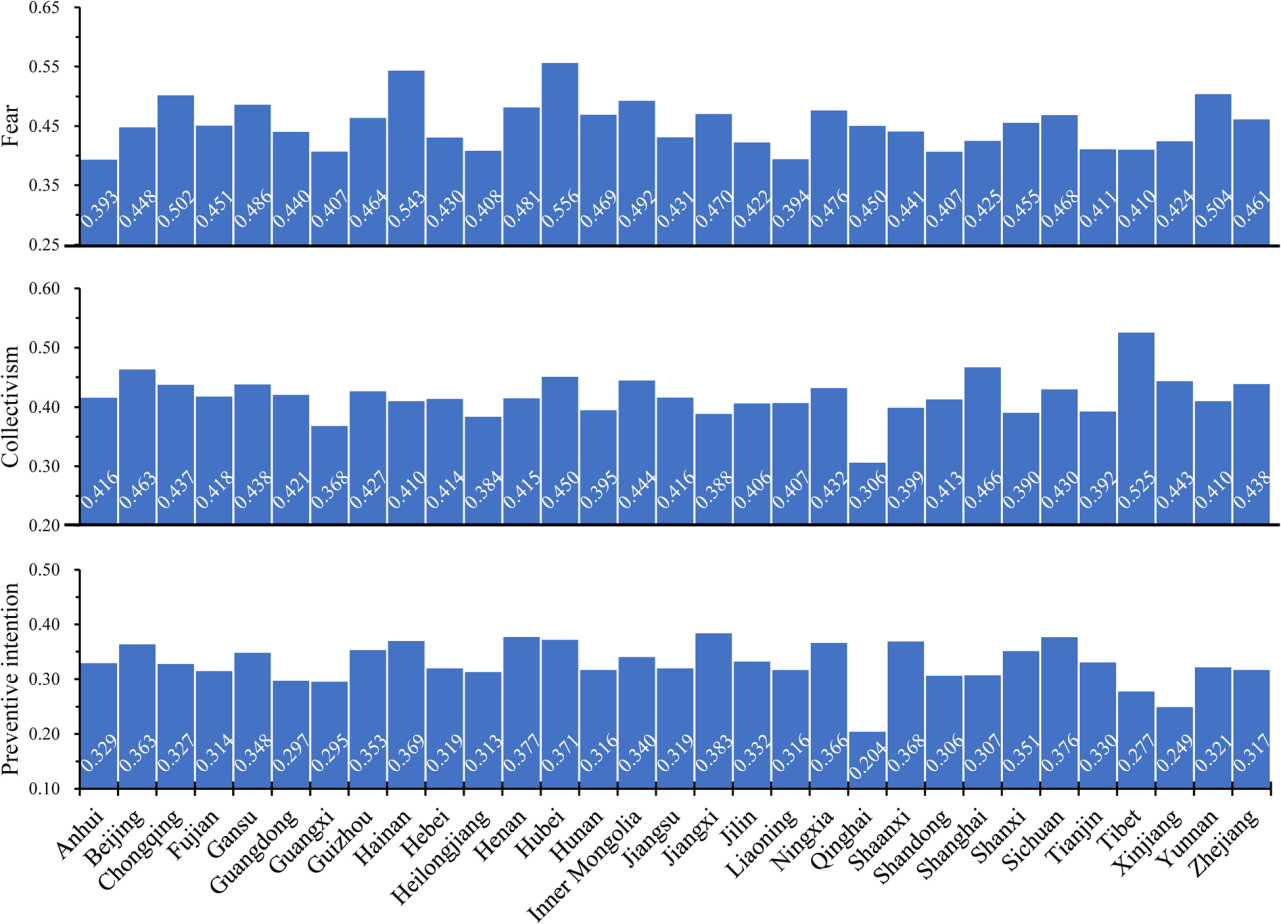
The means of fear, collectivism, and preventive intention during the outbreak in 31 regions of the Chinese mainland

We calculated the score of fear, collectivism and preventive intention score in 31 provinces by the day. In this study, the score of collectivism in the south is generally more significant than that in the north, which is consistent with the previous research of cultural psychology [20]. The average value of fear, collectivism and preventive intention in each province are shown in Fig. 3.

The *Pearson correlation analysis* shows that: (a) fear is positively correlated with collectivism and preventive intention (***p*** *<* 0.001); (b) there is a positive correlation between collectivism and preventive intention (***p*** < 0.001). The descriptive statistics and correlation coefficient of all variables are listed in Table 1.

**Table 1:** Descriptive statistics and correlation coefficient (***N*** = 1302)

Variables	***M***	***SD***	Fear	Collectivism	Preventive intention
Fear	0.452	.235	1		
Collectivism	0.428	.102	.202^***^	1	
Preventive intention	0.328	.261	.351^***^	.326^***^	1

Legend: *SE* standard deviation, *M* mean value

*** indicate statistically significant *p* values < 0.001

### The interaction of fear and collectivism on the preventive intention

A multiple regression model was established by the *PROCESS for SPSS*. Fear and collectivism, which are used to predict preventive intention, are the first to enter the equation. The results show that both fear and collectivism can positively predict the preventive intention (fear: ***β*** = 0.324, ***t*** = 11.685, ***p*** < 0.001; collectivism: ***β*** = 0.284, ***t*** = 10.433, ***p*** < 0.001). The Fear × Collectivism was then incorporated into the model to predict preventive intention jointly, the results show that the Fear × Collectivism regression coefficient is significantly (***β*** = − 0.134, ***t*** = − 5.963, ***p*** < 0.001). The regression models are summarised in Table 2.

**Table 2:** The interaction of fear and collectivism on preventive intention

Variable	Model 1		Model 2	
	***β***	***t***	***β***	***t***
Fear	0.324	11.685^***^	0.323	11.810^***^
Collectivism	0.284	10.433^***^	0.293	10.907^***^
Fear × Collectivism			-0.134	-5.963^***^
***ΔR***^**2**^	0.190		0.211	
***F***	153.549^***^		116.942^***^	

Legend: *** indicate statistically significant *p* values < 0.001

To explain the interaction of fear and collectivism on preventive intention clearly, we divided the collectivism and fear into high and low groups according to the mean value of ±1SD and conducted simple slope analysis.

**Fig. 4:**
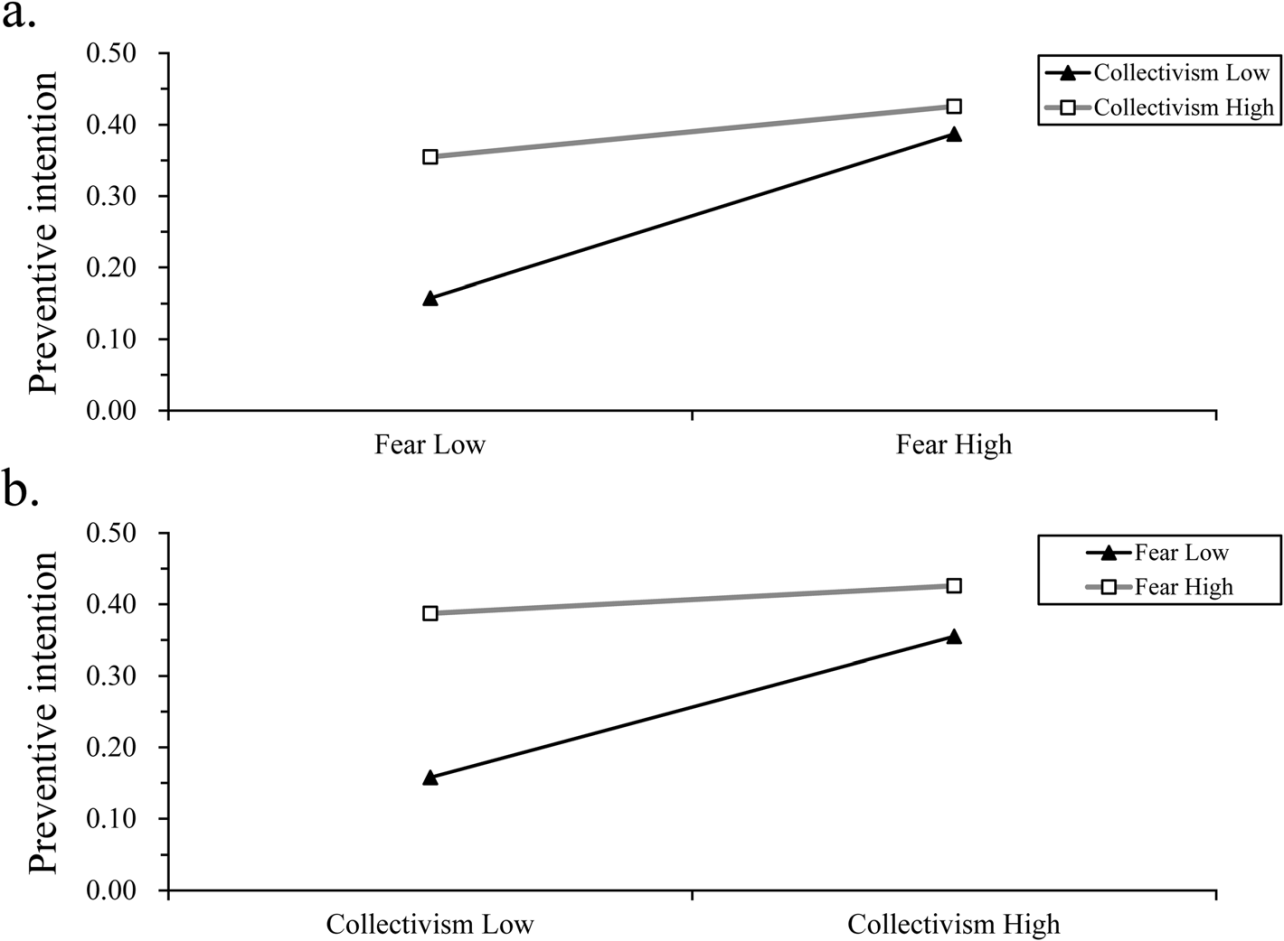
The interaction of fear and collectivism on preventive intention (simple slope analysis)

The diagrams of simple effect are shown in Fig. 4. Figure 4(a) show us, the fear positively predict preventive intention in low-collectivism group (***β*** = 0.457, ***t*** = 12.944, ***p*** < 0.001), while the predictive power of fear to preventive intention declines in high-collectivism group (***β*** = 0.189, ***t*** = 5.334, ***p*** < 0.001; *β* drops from 0.457 to 0.189). According to Fig. 4(b), when fear is low, collectivism positively predict preventive intention (***β*** = 0.404, ***t*** = 12.038, ***p*** < 0.001), while the predictive power of collectivism to preventive intention declines at a higher level of fear (***β*** = 0.141, ***t*** = 3.901, ***p*** < 0.001; ***β*** drops from 0.404 to 0.141).

**Fig. 5:**
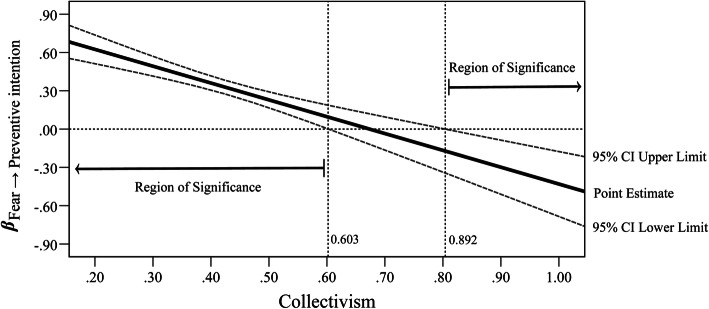
The interaction of fear and collectivism on preventive intention (Johnson-Neyman analysis)

The Johnson-Neyman technique was performed to explore the critical value of a significant regression coefficient. The results show that: (a) the standardised coefficient ***β*** of fear on preventive intention is significant when the collectivism is lower than 0.603, and the ***β*** decreases with the increase of collectivism; (b) when collectivism is in the range of [0.603, 0.892], the ***β*** is not significant; (c) When collectivism is higher than 0.892, the ***β*** changes from positive to negative and increases with the increase of collectivism. The Johnson-Neyman slope was plotted in Fig. 5.

## Discussion

To explore the combined effects of fear and collectivism on people’s preventive intention towards COVID-19, the present study analysed the Sina Microblog texts of 108,914 people. By using multivariate linear regression analysis, we found that there is an interaction between fear and collectivism on people’s preventive intention towards COVID-19. Fear and collectivism reduce each other’s positive influence on people’s preventive intention. Furthermore, a Johnson–Neyman slope test shows that when collectivism exceeds a certain level, the predictive effect of fear on people’s preventive intention towards COVID-19 shifts from positive to negative.

The positive relationship between collectivism and preventive intention in this study verifies the role of culture in the epidemic outbreak as speculated in the pathogen epidemic hypothesis. An investigation during the COVID − 19 pandemic showed that the facemask use rate measured in airport settings was the highest in Asia (46%) and the lowest in the USA (2%) [5], This may be related to the local cultural orientation, previous studies of cross-cultural psychology have shown that compared with western societies where individualism prevails, especially in the USA, Asia is more inclined to collectivism [48, 49].

The interaction of fear and collectivism on people’s preventive intention can be explained by combining the pathogen epidemic hypothesis and the cognitive resource theory. A previous study has found that collectivism can provide people with adequate psychological protection, thus enabling people to resist better negative emotions caused by disasters such as epidemics [28]. Correspondingly, the cognitive resource theory indicated that the psychological action of resisting negative emotions consumes a lot of cognitive resources [50].

During the COVID-19 pandemic, the people’s resistance to fear and the improvement of their preventive intention towards COVID-19 are both considered cognitive activities. Such activities need to consume or invoke cognitive resources, including collectivism. Conversely, higher collectivism helps people resist fear better, thus reducing the promoting effect of fear on their preventive intention towards COVID-19. Conversely, the increase of fear consumes more cognitive resources, including collectivism. Therefore, the promoting effect of collectivism on the preventive intention of COVID-19 is reduced. In sum, collectivism and fear weaken each other’s role in the promotion of people’s preventive intention towards COVID-19.

It is worth noting that people who are high in collectivism may not adopt more prevention measures because of the higher effectiveness of mental protection. Furthermore, the increase of fear will consume more cognitive resources and shall reduce people’s preventive intention accordingly. When people have already built a psychological defence system towards the COVID-19 pandemic, the increase of fear shall have negative influences on both the preventive intention and mental health of people.

Although fear in some cases is a limited contributor to people’s preventive intention, we should not ignore its adverse effects on both individuals and society. Previous studies have found that people’s fear is significantly and positively correlated with harmful psychological conditions, such as depression and anxiety [51]. During the COVID-19 pandemic, many people became suspicious that they were infected by the virus and therefore took their own lives—despite the autopsy results showing that they were physically normal [52, 53]. Negative emotions such as fear may devastate people’s mental health, so we recommend that governments take timely measures to deal with negative public psychology during and after the COVID-19 pandemic, such as to use cognitive behavior therapy to treat public’s depression and anxiety [54].

The COVID-19 pandemic has caused substantial negative impacts on many countries and regions around the world [55]. A common concern lies in the promotion of people’s preventive intention and pushing people to maintain a positive psychological state. Based on the social media big data of 108,914 active Microblog users, the study finds that the interaction of collectivism values and fear indeed affects people’s behavioural intentions. The results have some implications for the current pandemic control work.

The promotion of fear on people’s preventive intention may be limited and conditional, and values of collectivism can well compensate for the promotion of fear on preventive intention. Since fear may have a lasting negative impact on individuals, it is not advisable to promote preventive intention among people by arousing fear. On the one hand, we call on governments to timely dispel people’s fear and reduce social panic. On the other hand, we suggest that relevant departments conduct targeted publicity to promote people’s preventive intention through the establishment of a collectivist concept of health. For example, ‘Keeping social distance can protect you and your family from infection’ may be a more effective communication strategy than ‘Keeping social distance can protect you from infection’.

Our research has certain limitations, as well. First, there may be a sampling error. Among the users of Microblog, there are more young people than older people, and most of the participants came from urban areas instead of rural areas. Second, we only considered the cultural heterogeneity within China. The cross-cultural generalisation of the conclusion needs to be further verified. Third, the current analysis and interpretation are limited to a correlation level only. The causal relationship between variables cannot be determined yet. Further studies can build more sophisticated models to explore the causal relationship between cultural values, fear, and the prevention intention towards pandemics.

## Conclusion

By analysing the Sina Microblog data of 108,914 users in mainland China during the COVID-19 pandemic, the present study discussed the combined effect of fear and collectivism on the public’s preventive intention towards COVID-19. The findings are as follows: (a) both fear and collectivism can positively predict the people’s preventive intention and (b) fear and collectivism reduce each other’s positive influence on the people’s preventive intention. Considering the potential negative impact of fear on the individuals, the present study proposes to raise the public’s intention to curtail the spread of COVID-19 through collectivist propaganda.

## Data Availability

Due to protect the privacy of the participants, the original posts used for the analysis are not publicly available but are available from the corresponding author on reasonable request. The secondary data and code have been uploaded to the Open Science Framework (OSF) website and can be download through the following link: https://osf.io/mxrpn/?view_only=0720df84afc34c36b24c478935e571c4.

## Abbreviations

COVID-19: Corona Virus Disease 2019
PANAS: Positive and Negative Affect Schedule
ICS: the Individualism–Collectivism Scale
UISAQ: the Users’ Information Security Awareness Questionnaire
